# Non‐linear association between air pollutants and secondary sensitive skin in acne patients

**DOI:** 10.1111/jocd.16487

**Published:** 2024-07-26

**Authors:** Xiangfeng Chen, Jing Wen, Wenjuan Wu, Ying Tu, Qiuzhi Peng, Sifan Tao, Haoran Yang, Li He

**Affiliations:** ^1^ Department of Dermatology First Affiliated Hospital of Kunming Medical University Kunming China; ^2^ Liwa Institute of Skin Health East China Normal University Shanghai China; ^3^ School of Geographic Sciences East China Normal University Shanghai China; ^4^ Zhejiang Economic Information Center Hangzhou China; ^5^ Faculty of Land Resources Engineering Kunming University of Science and Technology Kunming China; ^6^ School of Mathematical Sciences East China Normal University Shanghai China; ^7^ Skin Health Research Center Yunnan Characteristic Plant Extraction Laboratory Kunming China

**Keywords:** air pollutants, machine learning, nonlinear relationships, sensitive skin secondary to acne, skin barrier

## Abstract

**Background:**

There is a growing number of patients suffering from sensitive skin secondary to acne, but its prevalence and influencing factors are not yet well‐understood.

**Objective:**

The aim of this study is to investigate the nonlinear relationship between air pollutants and secondary sensitive skin in acne patients.

**Methods:**

A cross‐sectional study comprising 4325 acne outpatients in China was carried out between September 2021 and December 2022, employing a simple random sampling approach. Air pollutants data was derived from the nearest air quality monitoring station corresponding to the subjects' residential locations. Furthermore, socio‐economic characteristics, biological attributes, and lifestyle data of patients were acquired via questionnaire surveys. The data were subsequently analyzed utilizing the XGBoost machine learning model.

**Results:**

A nonlinear relationship has been observed between secondary sensitive skin in acne patients and various factors, including particulate matter (PM_2.5_), inhalable particulate matter (PM_10_), ozone (O_3_), sulfur dioxide (SO_2_), nitrogen dioxide (NO_2_), carbon monoxide (CO), the severity of depression, different levels of exercise intensity, acne grading, frequency of sunscreen application, gender, and age.

**Conclusion:**

The occurrence of secondary sensitive skin in acne patients be mitigated through the implementation of measures such as the control of air pollutant emissions, regulation of negative emotions, and improvement of personal lifestyle.

## 1 INTRODUCTION

Acne, a chronic inflammatory skin condition affecting hair follicles and sebaceous glands, has been the subject of numerous epidemiological investigations both domestically and internationally. These studies have sought to examine the prevalence of acne and its associated influencing factors across diverse populations.^1^, ^2^ According to a systematic analysis conducted by the Global Burden of Disease Study in 2010, acne exhibited a prevalence rate of 9.38% within the global population, ranking it as the eighth most prevalent disease worldwide.^3^, ^4^ Following an episode of acne, sequelae including erythema, pigmentation, and scars are frequently observed,^5^, ^6^, ^7^ drawing significant attention due to their profound impact on the quality of life experienced by patients. In clinical practice, there is currently a rising prevalence of acne patients exhibiting sensitive skin, characterized as secondary sensitive skin in the context of acne. This condition manifests through subjective symptoms such as burning, stinging, itching, and a sensation of tightness when acne patients are exposed to physical, chemical, and psychological stimuli. Accompanying objective signs such as erythema, scales, and dilated capillaries may or may not be present.^8^ Research has indicated that the skin barrier of acne patients is impaired by factors such as air pollutants, UV radiation, low humidity, the administration of retinoic acid drugs, and negative emotions.^9^, ^10^, ^11^ However, there is currently a lack of epidemiological studies reporting the prevalence and influencing factors of secondary sensitive skin in acne.

Air pollutants is globally recognized as a health hazard,^12^, ^13^ The skin serves as the interface between the human body and the surrounding atmosphere, making it the primary contact surface for air pollutants. Common indicators of air pollutants encompass particulate matter (PM_2.5_), inhalable particulate matter (PM_10_), ozone (O_3_), sulfur dioxide (SO_2_), nitrogen dioxide (NO_2_), and carbon monoxide (CO).^14^, ^15^ Epidemiological investigations have revealed that various common skin diseases, such as specific dermatitis, acne, and psoriasis, are influenced by air pollutants,^16^, ^17^, ^18^ However, there has been no epidemiological investigation into the role of air pollutants in secondary sensitive skin in acne. The current comprehension of the connections between skin disease and air pollutant factors predominantly relies on linear hypotheses employing traditional statistical approaches; however, this may oversimplify the complex relationships between them.

In this context, 4325 valid questionnaires were collected from seven major geographic regions in China for the present study. The XGBoost machine learning method was employed to investigate the relationship between air pollutants, individual socio‐economic and biological factors, and the likelihood of secondary sensitive skin in acne.

## 2 METHODS

### 2.1 Research subject

**FIGURE 1:**
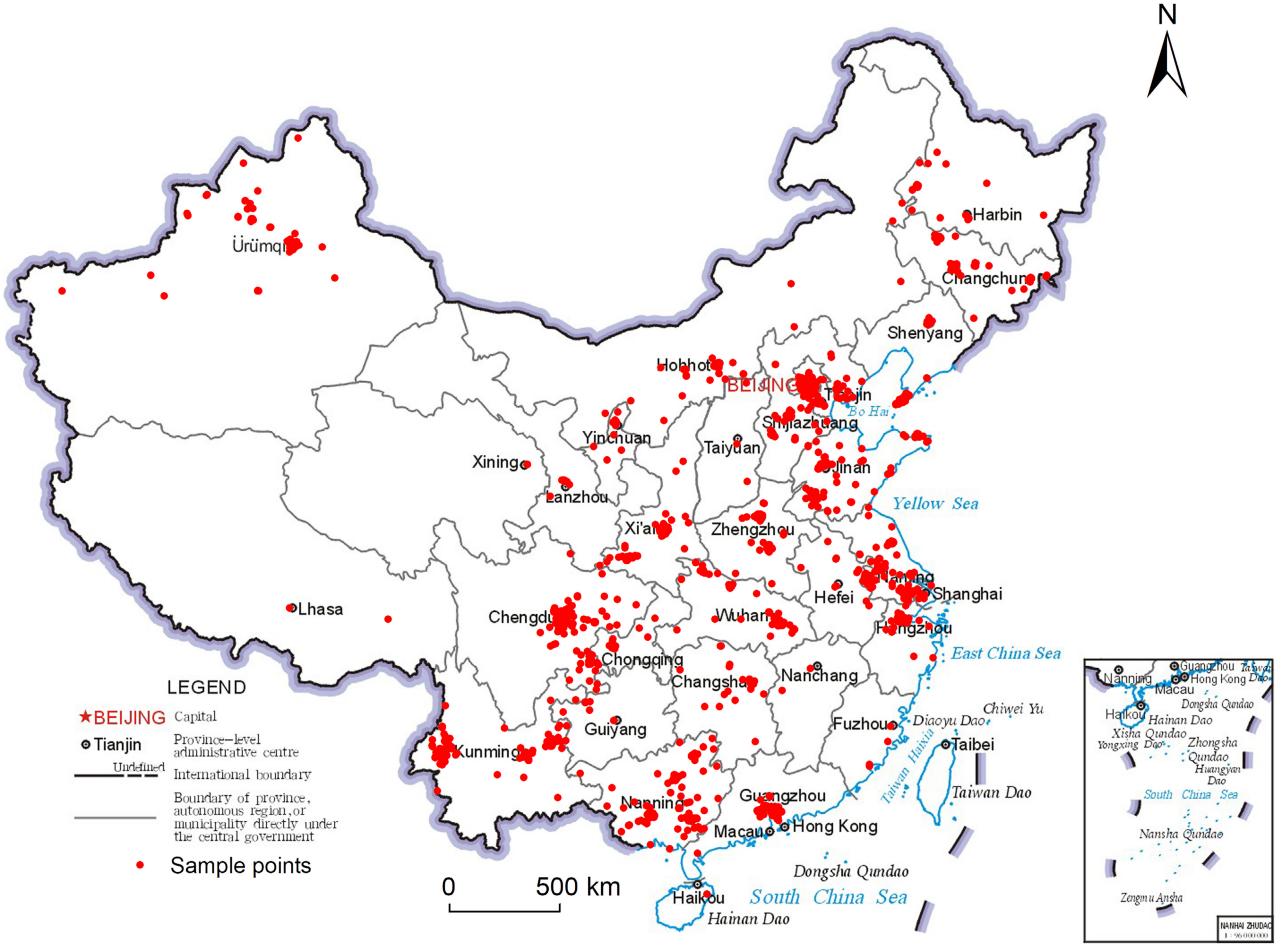
Sample distribution map for this study.

The personal information for this study was obtained from an epidemiological survey of secondary sensitivity skin to acne. The study protocol was approved by the Ethics Committee of the First Affiliated Hospital of Kunming Medical University, and all participants signed informed consent forms. A cross‐sectional study was conducted, involving acne patients from 70 hospitals located across seven major geographical regions in China, spanning the period from September 2021 to December 2022. The study employed a simple random sampling method. China has a vast territory and a wide latitude and longitude range. According to the geographical division of public health research, it can be divided into North China, Central China, East China, South China, Southwest, Northeast, and Northwest.^19^, ^20^ A total of 5019 subjects were included in the questionnaire survey, among which the case group was secondary sensitive skin to acne; The control group consisted of patients with only acne. Finally, after deleting data with missing and outliers, there were 4325 valid samples. (Figure 1).

Inclusion criteria:

- Between the ages of 12 and 55.
- Acne patients, judgment criteria: diagnosis by a dermatologist with clinical experience.
- Patients with sensitive skin, judgment criteria: Subjects who answer “yes” to at least five of the seven major questions in the table are considered sensitive skin,^21^, ^22^ otherwise they are considered non sensitive skin (Table S1)
- Informed consent for research.

The exclusion criteria are as follows:

- Pregnant, expectant, or lactating women.
- Local skin combined with other skin diseases: eczema, atopic dermatitis, contact dermatitis, psoriasis, and so forth.

In this study, residential addresses of 4325 valid samples were collected from China, and data were gathered from the nearest air quality monitoring station. The study encompassed six types of air pollutants: PM_2.5_, PM_10_, SO_2_, NO_2_, CO, and O_3_. The data comes from the national urban air quality real‐time release platform website of the China Environmental Monitoring Station from January 1 to December 31 in 2021, we extracted the 24‐haverage of pollutants and compiled it into the annual average for each station. The results pertaining to depression were assessed using the PHQ9 questionnaire, while lifestyle and personal socio‐economic attributes were acquired from the questionnaires completed by the participants.

### 2.2 Statistics

The baseline characteristics were analyzed using SPSS software, version20. The age distribution, denoted by *M* (*P*_25_, *P*_75_), does not conform to a normal pattern and was assessed for differences using a rank sum test. Family income, presented as ordered multiclass data in the form of *n* (%), was compared between groups utilizing rank sum tests. Gender, ethnicity, and marital status, all binary data expressed as *n* (%), underwent analysis for differences through chi‐square tests. A significance level of *p* < 0.05 indicates statistical significance in observed differences.

### 2.3 XGBoost model

It is well‐known that Random Forest (RF) and Gradient Boosting Decision Trees (GBDT) are classic models in ensemble methods. RF constructs individual trees using random samples of data and combines the results at the end,^23^ while GBDT sequentially builds trees and combines results throughout the process.^24^ Each model has its strengths and weaknesses. RF is suitable for large sample sizes, but given the sample size in this study, RF does not offer an advantage over GBDT and generally performs worse. XGBoost was proposed by Chen and Guestrin in 2016,^25^ It is a scalable system for efficiently implementing GBDT, addressing the overfitting issues commonly associated with GBDT. Moreover, the processing speed of XGBoost is over 10 times faster than other solutions for GBDT. Consequently, XGBoost has gained a strong reputation and popularity in data science.^26^

The algorithm of XGBoost primarily consists of three steps: data preprocessing, model training, and model prediction. First, data preprocessing is undertaken to clean and process the original data. This step includes handling outliers, selecting relevant features, and encoding features to ensure the quality of the data. Next, the model training phase is executed. XGBoost employs a decision tree‐based ensemble learning method.^27^ Initially, the model is initialized, and some initial parameters are set. The model is then continuously optimized through iterative processes. During each iteration, the gradient and second derivative of the loss function are calculated based on the current model's performance. This information is used to grow and prune the tree.^24^ Additionally, regularization terms and learning rate mechanisms are introduced to prevent overfitting and accelerate model convergence.^28^ Finally, the model prediction stage is conducted. The final prediction result is obtained by aggregating the predicted results of each tree. The decision‐making process of the model can be understood by examining the depth of the tree, the splitting characteristics of nodes, and the information on splitting points.

In this study, the XGBoost model was used to identify the relevant factors of sensitive skin in acne patients and their associations. In the present investigation, 70% of the data was randomly allocated to the training set, while the remaining 30% constituted the validation set. A five‐fold cross‐validation approach was also implemented. Parameters such as learning rate (eta), maximum tree depth (max_depth), and regularization terms (lambda and alpha) were fine‐tuned using a grid‐search algorithm. The optimal model parameters were found to be eta = 0.1, max_depth = 3, lambda = 0.5, and alpha = 1.

## 3 RESULTS

In this study, there were 4325 valid samples. One thousand four hundred twenty‐four individuals with secondary sensitivity to acne were identified and subsequently matched with 2811 control subjects. The overall prevalence of secondary sensitivity to the skin among the 4235 acne patients was found to be 33.6%. In the course of this investigation, no significant distinctions were discerned in age, ethnicity, marital status, and annual family income between the case group and the control group (*p* > 0.05). However, a gender disparity was observed between the two groups, with statistical significance (*p* < 0.05). The descriptive statistics for the features are presented in (Table 1).

**TABLE 1:** Baseline characteristics of sensitive skin secondary to acne patients and controls.

Baseline characteristics	Secondary sensitive skin in acne (*n* = 1424)	Acne (*n* = 2811)	*p* value
Gender, No (%)			
Male	259 (18.2)	853 (30.3)	<0.001
Female	1165 (81.8)	1958 (69.7)	
Age (years), Median (IQR)	24 (21,28)	24 (21,28)	0.164
Ethnicity, No (%)			
Han	1264 (88.8)	2520 (89.6)	0.378
Minority	160 (11.2)	291 (10.4)	
Marriage, No (%)			
Unmarried/single	1093 (76.8)	2139 (76.1)	0.632
Married	331 (23.2)	672 (23.9)	
Annual family income (￥), No (%)			
<80 000	687 (48.2)	1386 (49.3)	0.38
80 000–300 000	656 (46.1)	1290 (45.9)	
>300 000	81 (5.7)	135 (4.8)	

*Note*: Non‐normal distribution data are presented as median (interquartile range). **￥**is the symbol of RMB.

Abbreviation: IQR: interquartile range.

**FIGURE 2:**
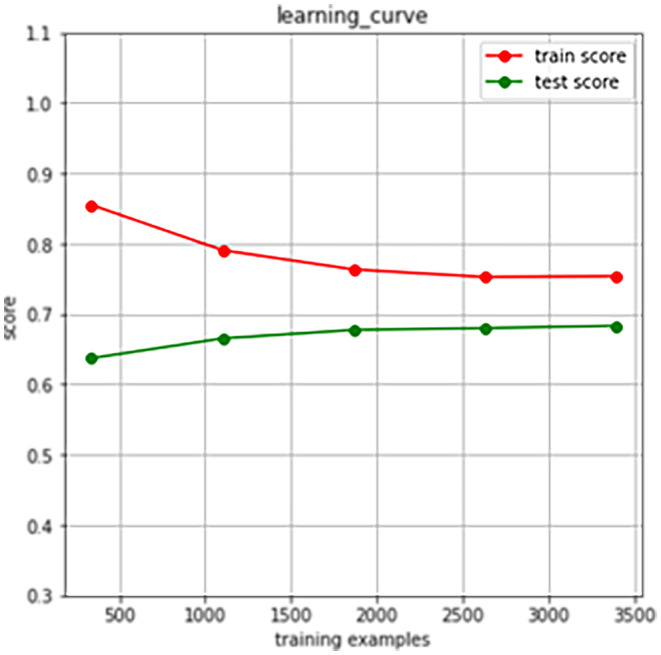
The learning curve of the xgboost model.

**FIGURE 3:**
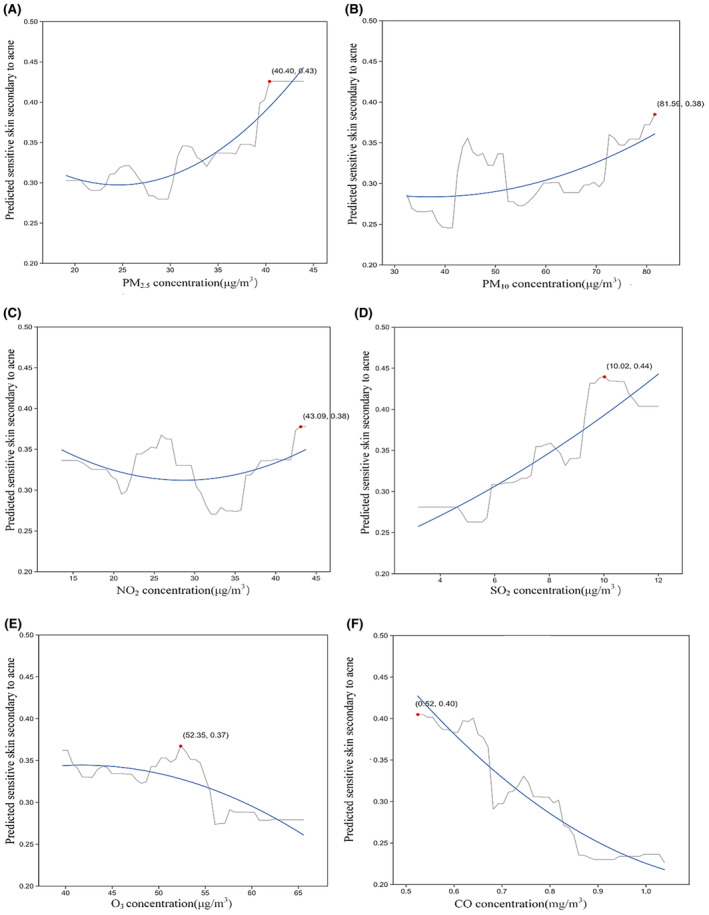
Nonlinear relationship between six common air pollutants and secondary sensitive skin in acne patients. (A‐F) CO, carbon monoxide; NO2, nitrogen dioxide; O3, ozone; PM10, inhalable particulate matter; PM2.5, particulate matter; SO2, sulfur dioxide.

**FIGURE 4:**
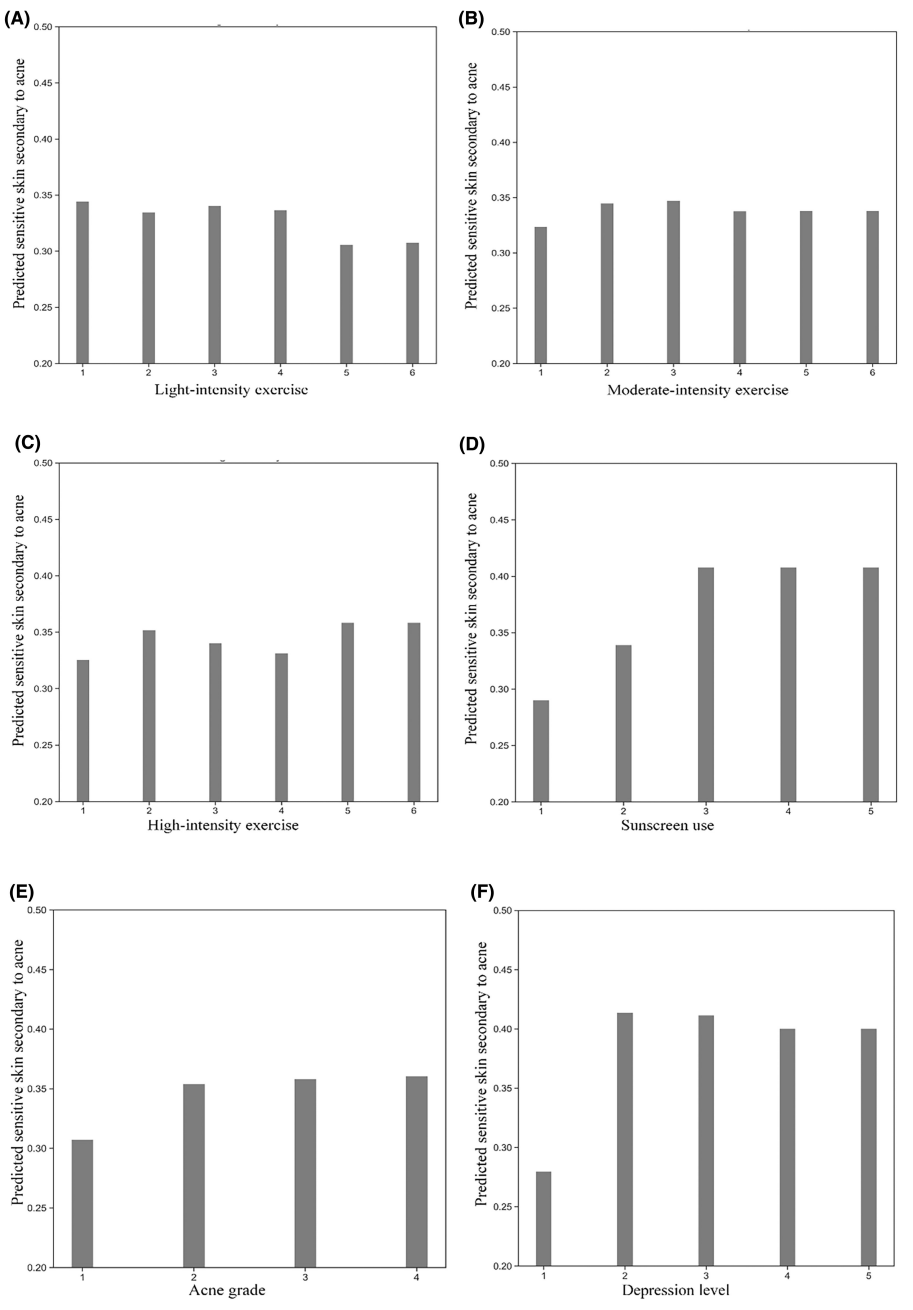
Nonlinear relationship between biological characteristics, lifestyles and secondary sensitive skin in acne patients. (A–C) The horizontal axis represents different intensities of exercise over time. The numerical value “1” on the horizontal axis indicates almost no exercise per week, “2” indicates less than 1 h of exercise per week, “3” represents one to 2 h of exercise per week, “4” represents 3–4 h of exercise per week, “5” represents 5–6 h of exercise per week, and “6” represents seven or more hours of exercise per week. (D) The horizontal axis represents the frequency of sunscreen usage. The numerical value “1” on the horizontal axis represents no use of sunscreen; “2” represents occasional use of sunscreen; “3” represents daily use of sunscreen once; “4” represents daily use of sunscreen two to three times; “5” represents daily use of sunscreen more than three times. (E) The horizontal axis represents the grading of acne. The numerical value “1” on the horizontal axis represents Grade I acne; “2” represents Grade II acne; “3” represents Grade III acne; “4” represents Grade IV acne. (F) The horizontal axis represents the level of depression. The numerical value “1” on the horizontal axis represents absence of depression, “2” represents mild depression, “3” represents moderate depression, “4” represents moderately severe depression, and “5” represents severe depression.

**FIGURE 5:**
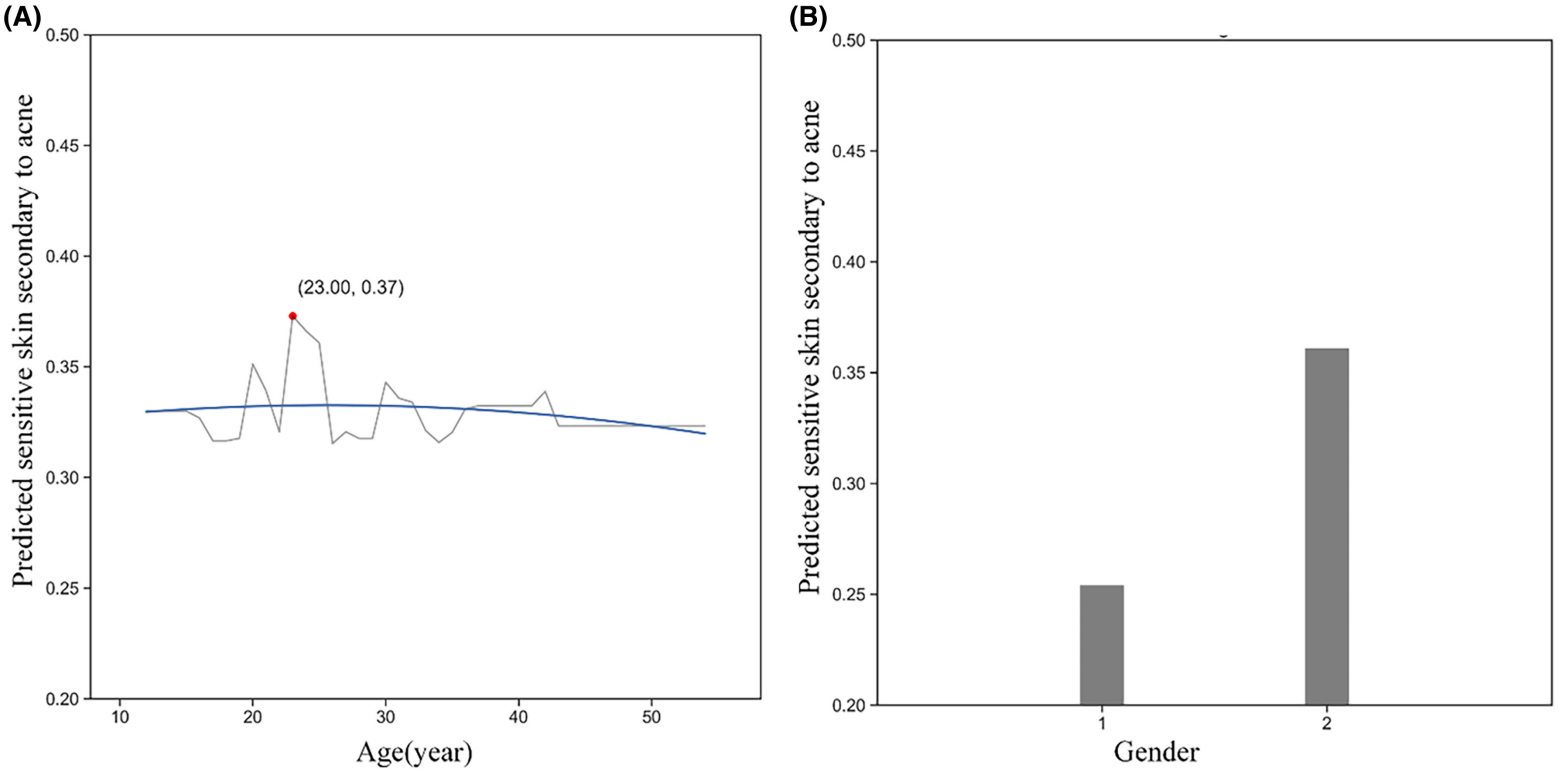
Nonlinear relationship between socioeconomic attributes and secondary sensitive skin in acne patients. (A) The horizontal axis represents age. (B) The horizontal axis represents gender. The numerical value “1” on the horizontal axis represents “male”, and “2” represents “female.”

The learning curve of our model suggests that as the number of data points increases, the training score tends to diminish while the test score rises. Both curves converge within a range of approximately 0.7–0.8, indicating the absence of overfitting due to the minimal discrepancy between training and validation errors (Figure 2). To facilitate the interpretation of machine learning models, Partial Dependence Plots (PDP) were employed to vividly visualize the marginal effects of these features on the outcome (Figures 3, 4, 5). In Figures 3, 4, 5, the x‐axes represent the independent variables, while the y‐axes denote the probability of secondary sensitivity to acne. Figure 3 illustrates the relationships between sensitivity skin secondary to acne patients and six common air pollutants. In Figure 4, we provided a detailed analysis of the complex relationships between biological characteristics, lifestyles, and the occurrence of secondary sensitive skin in individuals with acne. In Figure 5, we presented an analysis of the relationships between socioeconomic attributes and the occurrence of sensitive skin secondary to acne.

## 4 DISCUSSION

Presently, research suggests that the occurrence of sensitive skin is associated with four major mechanisms, namely skin barrier damage, neurohyperresponsiveness, vasodilation, and inflammatory response.^29^, ^30^ Various internal and external factors can result in damage to the skin barrier in acne patients, heightened reactivity of their nerve endings, induction of skin vascular dilation, or elicitation of inflammatory reactions, thereby mediating their sensitivity.

As shown in Figure 3, as the concentration of PM_2.5_ increases, the risk of secondary sensitivity to acne in patients also rises. Once the concentration reaches 40 μg/m^3^, the risk experiences a sharp increase, followed by stabilization. In human keratinocytes, PM_2.5_ has been observed to downregulate silk fibroin by upregulating the expression of cyclooxygenase 2 (COX2) and prostaglandin E2 (PGE2).^31^, ^32^ When subjected to PM_2.5_, both human and mouse skin equivalents exhibit an inhibition of fibronectin expression and an increase in transdermal water loss, ultimately resulting in the impairment of the skin barrier.^33^ Additionally, the morphology and structure of keratinocytes are disrupted by PM_2.5_, leading to a disturbance in the skin barrier.^34^

Regarding PM_10_, when the concentration of PM_10_ falls within the range of 40 μg/m^3^ to 55 μg/m^3^, there is an initial increase in the risk of sensitivity secondary to acne patients, followed by a subsequent decrease. Once the concentration surpasses 55 μg/m^3^, the likelihood of the disease exhibits fluctuations. It has been observed in research that mice subjected to PM_10_ exposure experience a notable elevation in epidermal water loss (TEWL) via the skin. In the case of healthy human skin, an escalation in PM_10_ concentration may also result in compromised barrier function.^35^

In terms of NO_2_, as the concentration of NO_2_ increases, the risk of sensitive skin secondary to acne fluctuates. The risk reaches its lowest point at a concentration of approximately 32 μg/m^*3*^. After this point, the risk gradually increases again. Previously, it has been found that exposure to NO_2_ increases the TEWL value of skin, leading to damage to the skin barrier.^36^

As the concentration of SO_2_ increases, the risk of sensitive skin secondary to acne gradually increases in fluctuations when the concentration of SO_2_ reaches 10 μg/m^3^, the risk of sensitive skin secondary to acne is predicted to be at its highest. This suggests that high levels of SO_2_ in the environment can significantly impact the occurrence of sensitive skin secondary to acne.

When the concentration of O_3_ falls within the range of 40–52 μg/m^3^, a higher likelihood of experiencing secondary sensitivity to acne is observed among individuals, as the concentration ranges from 52 to 56 μg/m^3^, the risk rapidly decreases, Moreover, once the concentration exceeds 56 μg/m^3^, the probability of illness remains at a relatively low and stable level. It has been shown that O_3_ exposure leads to various skin inflammation through redox pathway,^37^ At the same time, O_3_ therapy can be applied to the clinical treatment of various inflammatory skin.^38^, ^39^

For CO, as the concentration increases, the risk of sensitive skin secondary to acne drop rapidly with some fluctuations, Indicating that CO is a protective factor for acne patients with secondary sensitive skin. The anti‐inflammatory effect of low concentrations of CO supports this viewpoint.^40^ Low concentrations of carbon monoxide can lead to the inhibition of tumor necrosis factor‐α and interleukin‐1β, as well as decrease of interleukin‐10, thereby reducing the inflammatory response.^41^

In Figure 4, as a low‐energy‐consuming form of physical activity, mild‐intensity exercises like jogging or yoga exhibit a decrease in the incidence of secondary sensitivity to acne as exercise duration increases. Once the weekly exercise time reaches 5–6 h (represented by the horizontal axis value of 5), the risk of secondary sensitivity to acne remains essentially unchanged. In the context of moderate‐intensity exercise, the highest prediction of secondary sensitivity to acne in patients is observed when 1–2 h per week is considered (represented by the horizontal axis value of 3). As exercise time is further extended, no significant alteration in the risk of secondary sensitivity to acne in patients is noted.

Furthermore, when high‐intensity exercise is undertaken by acne patients for 5–6 h per week (represented by the horizontal axis value of 5), the highest risk of developing secondary sensitivity to acne is observed. This heightened risk is then maintained at a relatively elevated level. Previous studies have established that the perception of various stimuli within the human body can undergo alterations during and after exercise, including a reduction in pain sensation, which is associated with the activation of opioid substances induced by exercise,^42^ The thermal sensitivity function of the limb skin can be reduced after high‐intensity exercise.^43^ Our research results confirm that as the duration of mild intensity exercise increases, the protective effect on secondary sensitive skin to acne becomes stronger.

As patients transition from not using sunscreen at all to occasionally using sunscreens once a day, the risk of developing acne‐prone sensitive skin gradually increases. However, as they increase the frequency of sunscreen application to twice or three times a day, the probability of developing sensitive skin remains stable. The results do not match expectations. Previous studies have shown that exposure of human keratinocytes to UVB can damage the hydration of the stratum corneum.^44^ Irradiation of hairless mice with UVB revealed increased epidermal thickness, TEWL value, decreased water content in the stratum corneum, and other epidermal barrier dysfunction,^45^, ^46^ As we all know that sunscreen use provides ultraviolet radiation (UV) protection,^47^ Hence, there is a need for an improvement in the criteria employed for the formulation of cosmetics, as numerous chemicals, either individually or in combination, have the potential to be deemed unsafe. Research has shown that increased exposure to ultraviolet (UV) rays, such as during vacation, leads to more frequent use of sunscreen compared to periods of work.^48^ However, when the damaging effects of UV rays on the skin barrier outweigh the protective effects of sunscreen, the skin is more likely to develop secondary sensitivity. To better understand the factors influencing secondary sensitivity in acne‐prone skin, future research will focus on collecting data on subjects' UV exposure and analyzing it in conjunction with their sunscreen usage.

Acne can be classified into three degrees and four levels based on its severity. Our study found that as the grading of acne increases, patients are more likely to develop secondary sensitive skin. When acne is classified as Grade IV, the risk of secondary sensitive skin is highest. First, patients with acne exhibit elevated Transepidermal Water Loss (TEWL) values, reduced skin conductance within the stratum corneum, and impaired hydration. These conditions render their skin more susceptible to the development of secondary skin sensitivities.^49^ Second, drugs used in the treatment of acne, such as benzoyl peroxide and isotretinoic acid, can also disrupt the epidermal barrier.^50^, ^51^ On the other hand, photodynamic therapy commonly used for moderate to severe acne can improve skin barrier function,^52^, ^53^ therefore, the impact of acne grading on the risk of secondary sensitive skin is not a simple linear relationship.

We use the PHQ‐9 scale, which stands for Patient Health Questionnaire‐9, to measure depression scores and levels (Table S2). When depression is absent among acne patients, they are less inclined to develop secondary sensitive skin. However, upon being diagnosed with depression, the risk of secondary sensitive skin increases rapidly and subsequently maintains a relatively high level. The presence of depression in these patients seems to exacerbate the likelihood of their skin becoming more prone to sensitivity. Previous studies have found that emotional changes are a stimulating factor for sensitive skin.^54^ Depression is associated with sensitive skin with facial flushing as the main clinical manifestation.^55^ Our study confirms the nonlinear relationship between depression and sensitive skin secondary to acne.

In Figure 5, as age increases, the risk of secondary sensitivity to acne remains stable with fluctuations. Notably, there is a dearth of epidemiological research investigating the influence of age on secondary sensitivity to acne. Experimental findings have indicated that with advancing age, human skin tends to become more acidified, resulting in heightened activity of TRPV1 due to lower PH values,^56^ the activation of TRPV1 leads to the influx of ions in sensory fibers, which then generates action potentials, ultimately leading to burning pain or itching.^57^ Meanwhile, the skin nerve innervation function related to touch and pain in the human body will decrease with age,^58^ The decrease in sensory nerve function and reduced skin innervation may offset the age‐induced increase in TRPV1 expression.^59^

Female acne patients are more likely to develop sensitive skin than men. This may be due to the significantly higher thickness of the male epidermis and dermis,^60^ resulting in lower skin permeability and less susceptibility to stimuli or allergens. On the other hand, it may also be related to female hormonal fluctuations leading to skin sensitivity.^61^

This study extracted the longitude and latitude of the residential address of 4325 subjects and collected data from the nearest air pollutant site to explore the impact of air pollutants on secondary sensitive skin to acne. Our findings offer valuable insights into preventive strategies for secondary sensitivity to acne and serve as a theoretical foundation for measures aimed at preventing and treating air pollutants. Our sample encompasses seven major geographical regions in China, exhibiting both regional variations and representativeness. Furthermore, we investigated the nonlinear relationship between feature variables and the probability of secondary sensitivity to acne by employing the xgboost model in machine learning.

There are several limitations in our research. First, this study is cross‐sectional, which allows us to establish correlations between air pollutants, personal socio‐economic attributes, and biological factors with secondary sensitivity to acne, but it does not establish causal relationships. Second, our study did not specifically investigate the impact of topical drug therapy, dietary factors, and other variables on secondary sensitivity to acne. Future research could build upon this study to explore these aspects in greater detail.

## 5 CONCLUSION

The mitigation of secondary sensitive skin in individuals suffering from acne can be achieved through the implementation of various measures. These measures encompass the control of air pollutant emissions, the regulation of negative emotions, and the enhancement of personal lifestyle. The implementation of these strategies is crucial for addressing the issue of secondary sensitive skin, thereby contributing to the overall well‐being of acne patients.

## AUTHOR CONTRIBUTIONS

All authors have read and approved the final manuscript. Conceptualization: X.F.C., H.R.Y., L.H. Methodology: X.F.C., J.W. Investigation: X.F.C., W.J.W., Y.T. Writing: X.F.C., J.W., H.R.Y., L.H., W.J.W., Y.T. Funding Acquisition: L.H. Supervision: L.H., H.R.Y. Formal Analysis: X.F.C., J.W., Q.Z.P., S.F.T.

## CONFLICT OF INTEREST STATEMENT

The authors state no conflict of interest.

## ETHICS STATEMENT

The personal information for this study was obtained from an epidemiological survey of secondary sensitivity skin to acne. The study protocol was approved by the Ethics Committee of the First Affiliated Hospital of Kunming Medical University, and all participants signed informed consent forms.

## Supporting information


Data S1.

## Data Availability

The data are available upon request by contacting the corresponding author (L H). However, in compliance with privacy regulations, information regarding respondents' current health status and personal details cannot be disclosed. The remaining data and supplementary files can be found in this paper.
