# Supplementary material for: Non‐linear association between air pollutants and secondary sensitive skin in acne patients
Source: J Cosmet Dermatol. 2024 Jul 26;23(12):4007–16. doi: 10.1111/jocd.16487 (PMC11626370; doi:10.1111/jocd.16487)
Supplement: Supplementary file 1 — Data S1. [file JOCD-23--s001.docx]

| **Supplementary Table1. Questionnaire for diagnosis of sensitive skin** |
| --- |
| 1. Would you say that your face/neck does not tolerate cold/hot weather or a cold/hot environment? |
| 2. Would you say that your skin face/neck does not tolerate rapid temperature changes? |
| 3. Have you already avoided the use of some cosmetic products that could, according to you, make your skin reactive? |
| 4. Have you already had an adverse reaction on your face/neck to a cosmetic or hygiene product? |
| 5. Would you say that your face/neck is reactive? |
| 6. Have you already felt some itching, burning or tingling on your face/neck skin because of the wind or some cosmetics or hygiene products? |
| 7. Is your face skin reactive to pollution, stress/emotions or menstrual cycle changes? |

Supplementary Table 2. Nine-symptom checklist of nine-item Patient Health Questionnaire (PHQ-9)

| **Over the last 2 weeks, how often have you bothered by any of the following problems?** | | **Not at all** | **Several days** | **More than half the days** | **Nearly every day** |
| --- | --- | --- | --- | --- | --- |
| Q1 | Little interest of pleasure in doing things | 0 | 1 | 2 | 3 |
| Q2 | Feeling down, depressed, or hopeless | 0 | 1 | 2 | 3 |
| Q3 | Trouble falling or staying asleep, or sleeping to much | 0 | 1 | 2 | 3 |
| Q4 | Feeling tired or having little energy | 0 | 1 | 2 | 3 |
| Q5 | Poor appetite or overeating | 0 | 1 | 2 | 3 |
| Q6 | Feeling bad about yourself-or that you are a failure or have let yourself or your family down | 0 | 1 | 2 | 3 |
| Q7 | Trouble concentrating on things, such as reading the newspaper or watching television | 0 | 1 | 2 | 3 |
| Q8 | Moving or speaking to slowly that other people could have noticed? Or the opposite-being so fidgety or restless that you have been moving around a lot more than usual | 0 | 1 | 2 | 3 |
| Q9 | Thoughts that you would be better off dead or hurting yourself in some way | 0 | 1 | 2 | 3 |
|  | | **Total score: ________________________** | | | |
